# Supercapacitors based on Ti_3_C_2_T_x_ MXene extracted from supernatant and current collectors passivated by CVD-graphene

**DOI:** 10.1038/s41598-020-80799-9

**Published:** 2021-01-12

**Authors:** Sunil Kumar, Malik Abdul Rehman, Sungwon Lee, Minwook Kim, Hyeryeon Hong, Jun-Young Park, Yongho Seo

**Affiliations:** 1 Department of Nanotechnology and Advanced Materials Engineering, Sejong University, Seoul, 05006 South Korea; 2 Graphene Research Institute and HMC, Sejong University, Seoul, 05006 South Korea

**Keywords:** Supercapacitors, Electrocatalysis

## Abstract

An ultrahigh capacity supercapacitor is fabricated using a nano-layered MXene as an active electrode material, and Ni-foil is used as a current collector. The high-quality Ti_3_C_2_T_x_ obtained from supernatant during etching and washing processes improves the specific capacitance significantly. As another strategy, the surface of Ni-foil is engineered by coating chemical vapor deposition-grown graphene. The graphene grown directly on the Ni-foil is used as a current collector, forming the electrode structure of Ti_3_C_2_T_x_/graphene/Ni. The surface passivation of the current collectors has a high impact on charge-transfer, which in turn increases the capacitance of the supercapacitors. It is found that the capacitance of the graphene-based supercapacitors is more than 1.5 times of the capacitance without graphene. A high specific capacitance, ~ 542 F/g, is achieved at 5 mV/s scan rate based on cyclic voltammetry analysis. Also, the graphene-based supercapacitor exhibits a quasi-rectangular form in cyclic voltammetry curves and a symmetric behavior in charge/discharge curves. Furthermore, cyclic stability up to 5000 cycles is confirmed with high capacitance retention at high scan rate 1000 mV/s. A reduced series resistance with a high limit capacitance is revealed by equivalent circuit analysis with the Nyquist plot.

## Introduction

MXene have been studied intensively as emerging inorganic materials with layered structures that have numerous applications, such as energy storage, fuel cells, 3D printing, conductive ink, EMI shielding, and supercapacitor^1–11^. MXene is a class of 2D compounds, which is obtained from transition metal compounds, known as MAX phases that have the general formula *M*_*n*+*1*_*AX*_*n*_, where M is generally an early transition metal (Ti, V, Nb, etc.), *A* is group 13 or 14 (mostly Al) element, X is C and/or N, and n = 1, 2, or 3^12^. MXene are produced by selectively etching out the *A* element from a MAX phase using HF or LiF/HCl etchant and forming the stacks of layered *M*_*n*+*1*_*X*_*n*_ structures, which are mostly carbides and nitrides. After etching of the MAX phases, the resultant MXene have some terminal groups, which include –OH, -O, -Cl, or -F, attached on the surface. Hence, the more general formula of MXene is represented as *M*_*n*+*1*_*X*_*n*_*T*_*x*,_ where *T* represents the terminal group^1,13^. Among them, Ti_3_C_2_T_x,_ is the most popular MXene that has been used in many applications, which include supercapacitors due to their high surface area and conductivity^7,14^. In order to improve the electrochemical properties of the MXene, various strategies have been adopted in the literature. These include the ions intercalation^3,15,16^, fabricating hybrid structures with graphene^17^ and doping^18^. Various attempts have been made in earlier reports to etch the MAX phases using the HF or the LiF/HCl based etchants for different applications^2,19–23^. However, the precipitated MXene have been dried and re-dispersed in water to obtain 2D structured MXene flakes in these reports. These exfoliated MXene are subsequently filtered and dried to obtain the paper-like free-standing electrodes that are used in supercapacitor fabrication.

The capacitance of supercapacitors is not only based on the active electrode materials, but it also depends upon the current collectors and electrolytes that are used. The capacitance of the supercapacitors can be improved by modifying the surface of the current collectors. There are some reports of the current collector surface passivation that have shown improvements in the electrochemical characteristics^24,25^. Owing to its astonishing properties, e.g. high surface area, electrical conductivity, and flexibility, graphene has been considered an essential material as electrodes of a supercapacitor^26,27^. It plays a role in passivating the current collector for supercapacitors and facilitates the contact between current collector and active electrode material. It has been previously reported that graphene passivation improved the capacitive properties when used with different active electrode materials^28,29^. The use of chemical vapor deposition grown graphene (CVD-G) coated Ni-foil as a current collector in MXene-based supercapacitors have not been reported.

In this report, we have attempted to improve the supercapacitor properties in two ways. First, the current collector, which is Ni-foil, was modified by coating its surface using CVD-G as Ni is an excellent catalyst for multilayer graphene growth as well as a good current collector. Second, ultrafine MXene structures were used, which were extracted by centrifuging the dark supernatant after the etching and washing processes. No report was found about the use of MXene structures extracted from the subsequent transfer of supernatants. The electrostatic charging properties of the supercapacitors based on this high-quality MXene have been studied as well as the effect of CVD-G passivation on the current collector surface.

## Materials and methods

**Figure 1:**
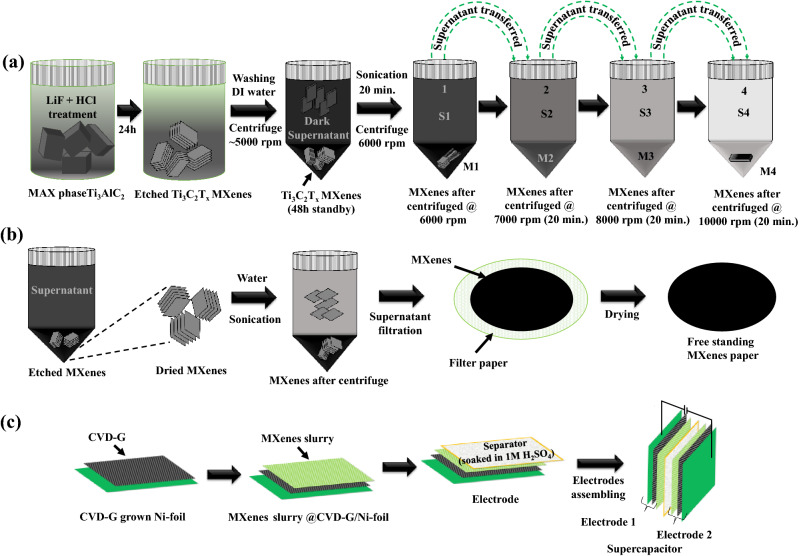
Ti_3_C_2_T_x_ MXene (**a**) synthesis from the supernatant by subsequent transfer in this study, (**b**) MXene synthesis in earlier studies for comparison, and (**c**) supercapacitor fabrication process.

The Ti_3_C_2_T_x_ MXene was obtained by etching Ti_3_AlC_2_ (> 98%, 200 mesh, Beijing Forsman Scientific Co., Ltd) using an LiF and 6 M HCl. 2 g LiF was mixed with 6 M HCl^2^. 2 g of Ti_3_AlC_2_ was added to this mixture, and it was stirred slowly for 24 h at room temperature (Fig. 1a). The resultant mixture was centrifuged and washed many times using DI water in order to be neutralized. During the washing process, it was observed that the supernatant did not become clear in spite of the repeated washings and the additional centrifugation at 4000–5000 rpm. The mixture in the tube was kept dormant for 48 h, but still the solution remained dark. Hence, a series of repeated centrifugation at relatively higher speed at each step with additional sonication was performed to obtain completely exfoliated MXene. Then, new MXene structures were extracted from this dark mixture, as illustrated in Fig. 1a. In the first step, the additional sonication and centrifugation were performed at ~ 6000 rpm for 20 min to obtain the high-yield precipitated MXene, which are called M1, and the dark supernatant called S1. S1 was transferred from Tube 1 to Tube 2, and Tube 2 was further centrifuged at ~ 7000 rpm for 20 min. The resultant precipitated MXene in Tube 2 were called M2, and the supernatant was called S2. This process was repeated, and the M3 were obtained from the S2 at ~ 8000 rpm centrifugation. The M4 was obtained from the S3 with the fourth centrifugation at ~ 10,000 rpm. For comparison, the MXene synthesis process adopted in earlier studies has also been depicted, which is shown in Fig. 1b, where a free-standing MXene paper was used as an electrode. On the other hand, in this study, the M1 and M4 in slurry form were coated on Ni-foils (thickness ~ 0.15 mm and an area 3 × 3 cm^2^) directly without using binder or carbon black. Two slurry-coated supercapacitor plates were dried at 100 °C and then assembled to form a two-electrode device by inserting a separator, which was a filter paper (Advantec MFS, Inc., USA) that had a thickness ~ 200 µm soaked in electrolyte 1 M H_2_SO_4_, between the two plates (Fig. 1c). The net weights of the active electrode material, which included M1 (~ 45 mg) and M4 (~ 4 mg), on both electrodes were estimated by measuring the mass differences before and after the MXene loading. As the M1 slurry included incompletely etched MXene, the loaded mass of M1 was considerably higher than M4. On the other hand, though a thin layer was coated with M4 MXene including completely etched MXene only on the Ni-foil, its mass was enough for experimental measurement as confirmed by repeated experiment.

The morphology and the structure of the etched Ti_3_C_2_T_x_ MXene were analyzed using field emission scanning electron microscope (FESEM, SU8010 Hitachi, Japan) and Raman microscopy (Renishaw, inVia, UK). The elemental composition of the Ti_3_C_2_T_x_ was confirmed using EDX spectroscopy, and the elemental distribution was determined using EDX mapping (EDX, Horiba). The composition and presence of the elements in the etched MXene were also confirmed using an x-ray photoelectron spectroscopy (XPS) analysis (K-alpha (Thermo VG, U.K.)) with a monochromated x-ray source (Al Kα line: 1486.6 eV). The surface area of the MXene were analyzed using the Brunauer–Emmett–Teller (BET) measurement technique (BELSORP-max, BEL Japan Inc.). The electrochemical studies were investigated using cyclic voltammetry (CV), galvanostatic charge–discharge (GCD), and electrochemical impedance spectroscopy (EIS), which were performed using potentiostat/galvanostat (Bio-logic, SP-150, USA). The sonication was performed using an ultrasonicator (Hwashin Technology Co. Ltd., Powersonic 410 (40 kHz), South Korea). The crystal structure of the MXene was analyzed using X-ray powder diffraction (XRD) system (PANalytical X’pert PRO).

## Results and discussion

**Figure 2:**
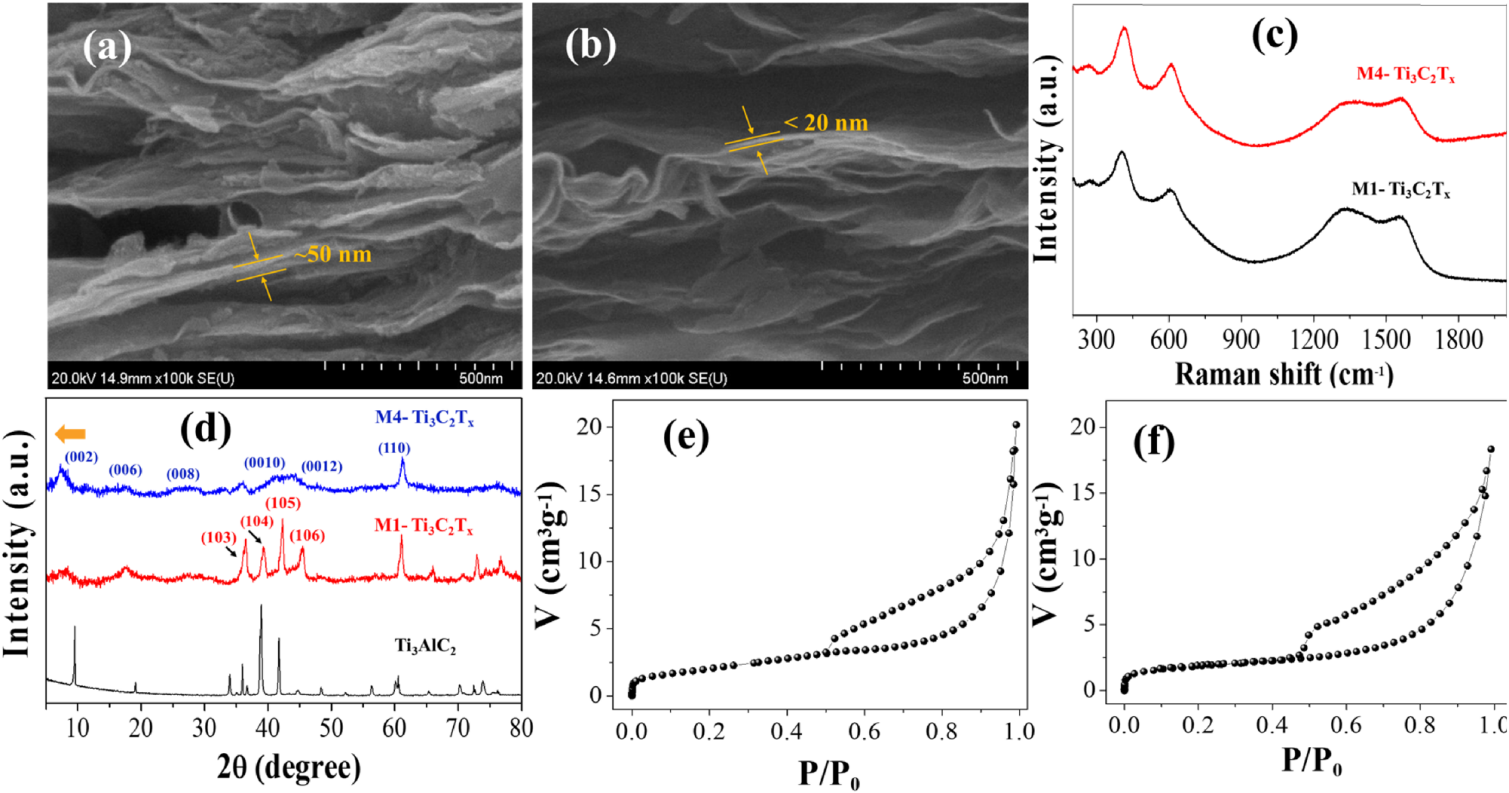
(**a**,**b**) FESEM images of M1 and M4 MXenes. (**c**) Raman spectra show characteristic peaks of Ti_3_C_2_. (**d**) XRD spectra were measured for M1 and M4. (**e**,**f**) BET curves of M1 and M4 MXenes are shown, respectively.

The morphologies of both types of fabricated Ti_3_C_2_T_x_ MXene and the CVD graphene grown on Ni-foil were investigated using FESEM. FESEM images of the graphene showed wrinkled morphology, which is mostly observed in the CVD graphene grown on Ni, shown in supplementary information Figure S1a. The FESEM images of the M1 and the M4 MXene are shown in Fig. 2a,b. The morphologies of the M1 and M4 MXene are layered as expected. It can be seen that the M4 MXene have nano-layers of thickness < 20 nm as compared to M1 MXene which has thickness > 50 nm. The elemental distributions in M1 and M4 MXenes were determined by EDX spectra as shown in Supplementary Information, Figure S2. Ti and C are obviously the expected elements, and F and O may be due to the terminal groups, whereas the negligible amount of Al is attributed to the incomplete etching or non-washed content, and the Cl may be due to impurities or the terminal group. The values of wt% and at.% are shown in Tables S1, S2, and S3 for Ti_3_AlC_2_ MAX phase, M1 and M4 MXene, respectively (Supplementary Information). It was observed that the most of Al is etched out as M1 MXene have 0.59 at.% Al, and M4 MXene have 0.17 at.% as compared to ~ 11 at.% in Ti_3_AlC_2_ MAX phase. The presences of Ti, C, and O were confirmed as major elements of Ti_3_C_2_T_x_ composition in the M1 and M4. In addition, the traces of F, and Cl were also found as elements of the terminal groups in both Mxenes.

The structural analysis of the CVD grown graphene on Ni-foil and M1, M4 MXene was performed using Raman spectroscopy. The graphene grown exhibited the characteristic G and 2D bands (Supplementary Information Figure S1b), which confirmed the growth of graphene. From the Raman spectra of the MXene (Fig. 2c), both Ti_3_C_2_T_x_ MXene have almost similar spectra, which indicates that the M1 and M4 Ti_3_C_2_T_x_ MXene have similar chemical structures. The spectra show peaks at ~ 200, ~ 390, and ~ 600 cm^−1^, which are characteristic peaks of Ti_3_C_2_ MXene. The peak ~ 200 cm^−1^ is associated with the Ti-C vibrations, whereas the peak ~ 390 cm^−1^ is associated with the O atoms vibrations. Another peak at ~ 600 cm^−1^ can be related to the E_g_ vibrations of the carbon in the Ti_3_C_2_T_x_ MXene that have –OH terminal groups^30^. There are two more peaks centered at ~ 1350 and ~ 1580 cm^−1^ that correspond to the D and G bands of the graphitic carbon, respectively^31^. The intensity of the D band is low compared to the G band which is according to the earlier report^32^.

The x-ray diffraction (XRD) patterns of Ti_3_AlC_2_ MAX phase, M1 and M4 MXene are shown in Fig. 2d. The XRD pattern of Ti_3_AlC_2_ exhibits XRD peaks (002), (004), (103), (104), (105) and (106) (JCPDS 52-0875). The XRD pattern of M1 MXene also exhibit the Ti_3_AlC_2_ like XRD peaks (002), (103), (104), (105) and (106), although low intensity as compared to Ti_3_AlC_2_, indicating that the M1 MXene still has some Al traces^19^. Judging from this M1 data, the crystal structure of M1 seems to have similarity to MAX, as Al was not completely removed. On the other hand, M4 MXene show characteristic peaks (002), (006), (008) and (110) at 2θ values, 6.9°, 17.4°_,_ 29.1° and 62.5°, respectively, exhibiting Ti_3_C_2_ signature peaks applications^19,33^. The peak (002) shifts to lower angle from MAX phase to M1, and it shifts further in case of M4 which indicates that the M4 MXene are more exfoliated as compared to M1 MXene^34^.

The surface areas of the MXene were estimated using the BET measurement by nitrogen adsorption and desorption isotherm at 77 K (Fig. 2e,f). The specific surface area of M1 and M4 MXene were found 9.887 m^2^/g and 13.245 m^2^/g, respectively, as estimated from the BET plots. Due to the more complete etching and exfoliation, the high surface area of M4 MXene was obtained.

**Figure 3:**
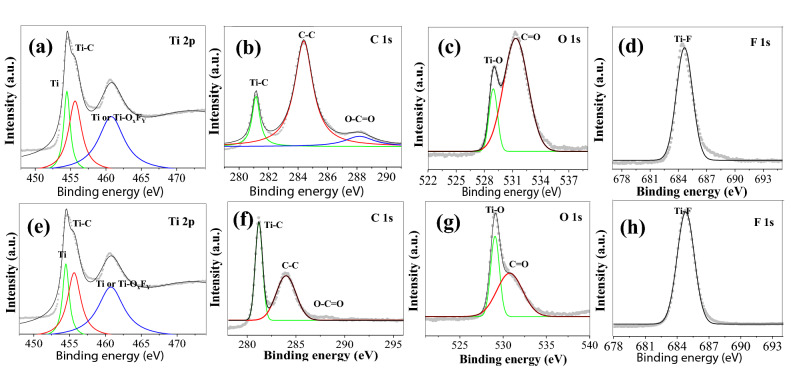
Highly resolved XPS spectra of Ti 2p, C 1 s, O 1 s, and F 1 s peaks in (**a**–**d**) M1 and (**e**–**h**) M4, respectively.

The presence of the elements and the composition of the synthesized M1 and M4 Ti_3_C_2_T_x_ MXene were confirmed using an XPS analysis. The complete XPS spectra of the M1 and M4 MXene are shown in Figure S3a,b (Supplementary information). The spectra of both MXenes have Ti 2p, O 1 s, C 1 s, F 1 s peaks, indicating that both MXene have Ti, C, O, and F as main elements. Beside these, a small intensity peak corresponding to Cl 2p was also observed in both MXene, indicating the presence of few Cl groups at MXene surface. Also, a very small intensity peak Al 2p was observed in both MXenes. Figure 3a–d, e–h are the high-resolution XPS spectra of Ti 2p, C 1 s, O 1 s, and F 1 s peaks present in M1 and M4 MXene, respectively. In Ti 2p spectrum, the peak at 454.7 eV due to the Ti 2p_3/2_ indicates the metallic Ti(II), whereas the peak at 455.7 eV indicates the Ti-C bond. Another peak at 460.8 eV may be due to either Ti-C or Ti-O_x_F_y_ in MXene (Fig. 3a,e)^35,36^. Similarly, in C1s spectrum, the peak at 281.9 eV, indicates the Ti-C bond, and the peaks at 284.2 eV indicate the C–C bond, whereas the peak at ~ 288 eV indicate O-C = O bond (Fig. 3b,f)^35^. In O 1 s spectrum, the peaks at ~ 528 and 532 eV indicating Ti–OH and C=O bonds, respectively, (Fig. 3c,g) may be associated with the O terminal groups^36^. In F 1 s spectrum, the peak at ~ 685 eV indicates a metal-fluoride bond (Fig. 3d,h), which may be a Ti-F bond, indicating the F as terminal groups attached in M1 and M4 MXene. Similarly, the peaks at 198 eV and 200 eV in Cl 2p spectra indicate the metal-chloride bond may be a Ti–Cl bond and organic chlorine (–C–Cl or –COCl), respectively (Fig. S3c,e, Supporting information). The high-resolution Al 2p spectrum has a peak in M1 MXene, centered ~ 75 eV, indicating the presence of Al in the form of aluminum oxide (Figure S3d, Supporting information). However, in case of M4 MXene, the peak of Al 2p spectrum, is not clear and only chaotic distribution was observed, indicating extremely low amount of Al in M4 MXene (Fig. S3f, Supporting information). The XPS analysis corroborates well with the XRD and the EDX results.

**Figure 4:**
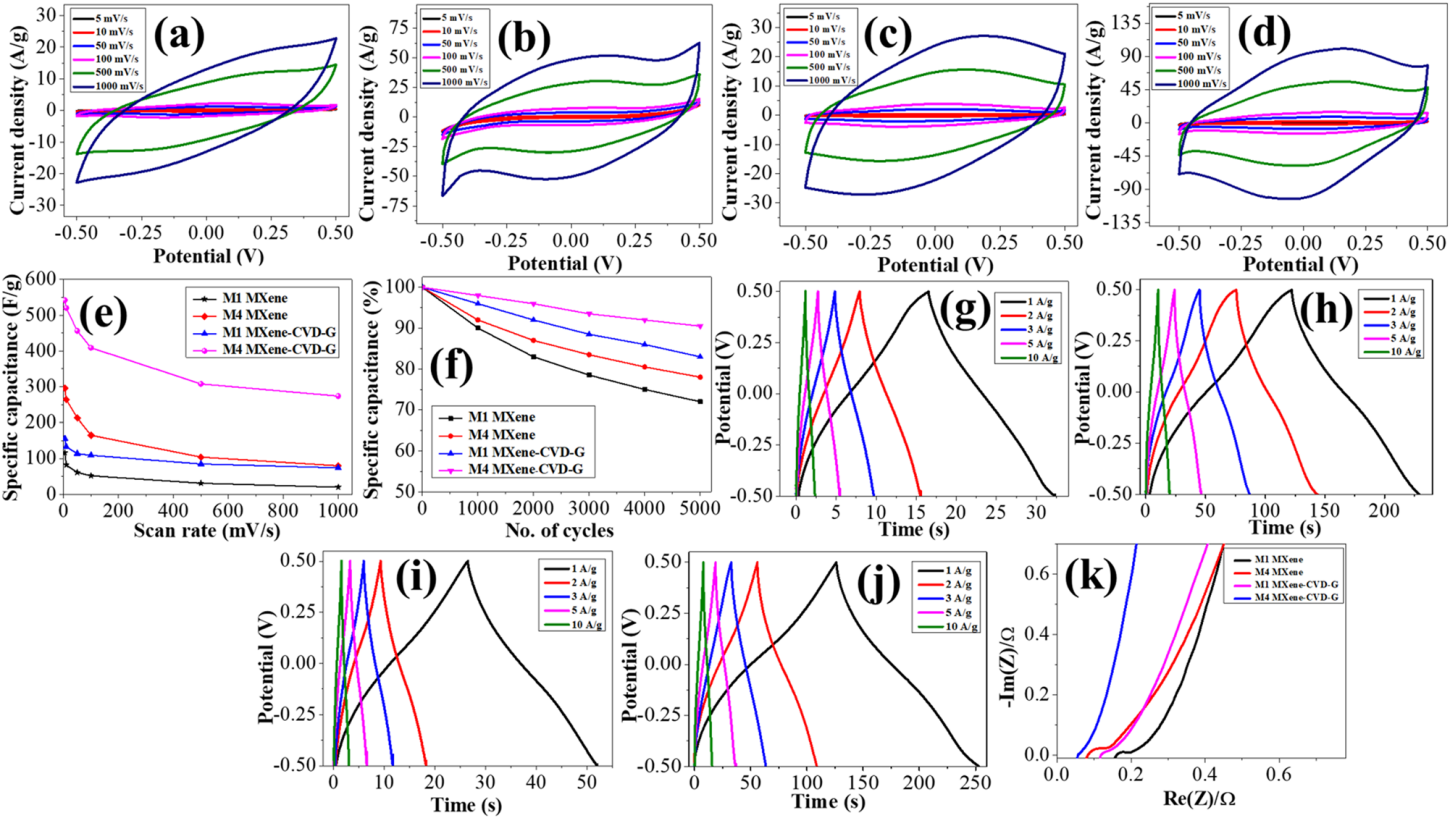
Electrochemical characteristics of the supercapacitors (**a**,**b**) CV curves without CVD-G and (**c**,**d**) with CVD-G for M1 and M4 MXene, respectively. (**e**) Capacitance retention at various scan rates. (**f**) Cyclic stability at 1000 mV/s till 5000 cycles. GCD curves without CVD-G (**g**,**h**) and with CVD-G (**i**,**j**) for M1 and M4, respectively. (**k**) Nyquist plots for M1 and M4 Ti_3_C_2_T_x_ MXene with and without CVD-G.

Figure 4 summarizes the electrochemical characteristics of the M1 and M4 MXene based supercapacitors. The specific capacitance (capacitance/g or C_g_) of the M1 and M4 MXene based two-electrode systems were estimated using CV plots recorded in a range from − 0.5 to 0.5 V at various scan rates ranged from 5 to 1000 mV/s (Slow scan rate magnified CV curves (5 and 10 mV/s) are shown in Supporting information Figure S6a–d). In this study, the purpose is to measure the capacitance in the potential window 1 V (− 0.5 to + 0.5 V) as it is known that for the aqueous acidic electrolytes the permitted voltage window is ~ 1.2 V. At a slow scan rate, 5 mV/s, the redox peaks were observed at around − 0.1 V and + 0.1 V indicating pseudo-capacitive behavior. Even at higher scan rates, such as 1000 mV/s the quasi-rectangular behavior was retained in all cases. However, it is more significant in the case of the CVD-G based supercapacitors. The area of the CV loops in the case of the M4 MXene is more than double compared to the M1 MXene (Fig. 4a–d), which indicates a higher capacitance. The specific capacitance of the supercapacitors was calculated using the following equation.

1 $${\text{C}}_{{\text{g}}} = \frac{{2\mathop \smallint \nolimits_{{v_{i} }}^{{v_{f} }} I dV}}{vVm},$$

where, *v*, *V*, and *m* are the scan rate, the voltage scan window, and the total weight of active electrode material on both electrodes, respectively, whereas $\int_{{v_{i} }}^{{v_{f} }} {I dV}$ is the area of the CV loop with *v*_*i*_ as the initial voltage and *v*_*f*_ as the final voltage.

The specific capacitances based on the CV curves, for the M1 and the M4 Ti_3_C_2_T_x_ MXene without CVD-G are: 116, 82, 61, 52, 31, and 20 F/g and 296, 264, 213, 165, 104, and 80 F/g at 5, 10, 50, 100, 500, and 1000 mV/s scan rates, respectively. The values of CVD-G based supercapacitors are: 156, 133, 114, 109, 85, and 74 F/g and 542, 520, 456, 409, 308, and 274 F/g at 5, 10, 50, 100, 500, and 1000 mV/s scan rates, respectively. It can be seen that the M4 MXene nearly have 2–3 times the specific capacitance as compared to the M1 MXene in both cases, which included with and without CVD-G, and it improves to more than 1.5 times when the Ni-foil current collectors are passivated with graphene. The higher specific capacitance in the M4 MXene may be associated with the higher surface area, proper interlayer spacing^37^, and the intercalated faradaic reactions within the MXene layers as well as at the MXene surface. It has been reported earlier that the major contribution to the capacitance in MXene supercapacitors using acidic electrolytes is due to the redox capacitance which involves the electrochemical adsorption of ions at the MXene surface and corresponding charge-transfer^38^. The specific capacitance of the M4 MXene and the CVD-G based supercapacitor is among the highest values obtained in the Ti_3_C_2_T_x_ based supercapacitors^3^. In earlier reports, the specific capacitances with LiF/HCl etched MXene were reported as 245 F/g and 370 F/g even at lower scan rate 2 mV/s^2,39^ for comparison.

The difference between the properties of M1 and M4 is mainly due to their etching. The M1 MXenes were not completely etched, less exfoliated, having comparatively lower surface area. For comparison, the specific capacitance of supercapacitors based on intermediate MXenes i.e. M2 and M3, were also calculated using CV curves (Figure S4a–d, Supporting Information), exhibiting similar trends either with or without CVD-G. The specific capacitances for M2 and M3 stand between those of M1 and M4 MXenes. The C_g_ of all MXenes calculated at 5 mV/s are shown in Table S4 (Supporting Information). The enhanced specific capacitance of CVD-G passivated supercapacitors can be associated with a reduced contact resistance between Ni-foil and MXene due to CVD-G^40^ and inhibition of direct reaction between acidic electrolyte and the metallic current collector. For comparison, the CV plots of Ni/CVD-G, Ni/M4, and Ni/CVD-G/M4 at 1000 mV/s scan rate are shown in Figure S4(i) (Supporting Information). The capacitance retention while moving from low to high scan rate i.e. 5–1000 mV/s is just ~ 20% in M1 and ~ 28% for M4 MXene without CVD-G, respectively. However, the capacitance retention improves to ~ 43% and ~ 52% in the M1 and the M4 MXene with the CVD-G, respectively (Fig. 4e). The cyclic stability of the fabricated supercapacitors was investigated at a high scan-rate of 1000 mV/s up to 5000 cycles. Even at high scan rate, after 5000 cycles the cyclic stabilities of the CVD-G based supercapacitors were maintained at ~ 91% for M4 and ~ 83% for M1, while those were ~ 78% and ~ 73% in the case of the MXene without CVD-G. (Fig. 4f). The lower cyclic stabilities may be associated with the absence of conductive carbon and binder. However, the CVD-G plays a role in maintaining the higher stability of the supercapacitors by passivating the Ni-foil.

The charge–discharge characteristics of the M1 and M4 Ti_3_C_2_T_x_ MXene based supercapacitors were estimated by analyzing the GCD curves between − 0.5 and 0.5 V at various constant current densities as shown in Fig. 4g–j. All the curves indicated a quick charge–discharge behavior and an almost negligible voltage drop even up to current density 10 A/g. The shape of the curves for the supercapacitors without CVD-G is less symmetric and triangular (Fig. 4g,h) as compared to the CVD-G based counterparts (Fig. 4i,j) in both types of Ti_3_C_2_T_x_ MXene, which indicates that CVD-G based supercapacitors exhibit better capacitor behavior^41^. A slight deviation from a perfect triangular behavior may be ascribed to the mainly pseudo-capacitive character in both MXene based supercapacitors^42^. The coulombic efficiency of M4 at 1A/g was estimated as ~ 0.8 due to a small leakage current, formation of solid electrolyte interface or electrolyte decomposition^43,44^. The charge–discharge curve based specific capacitance (C_g/cd_), specific energy density (E), and power density (P) were calculated using the equations^45,46^:

2 $$C_{g/cd} = 2 \times \frac{{{\text{I }} \times { }\Delta t}}{{{\text{m }} \times { }\Delta V}}$$

3 $$E = \frac{1}{2} \times C_{g/cd} \times \frac{{\Delta V^{2} }}{3600} \times 1000 \left( {{\text{Wh}}/{\text{kg}}} \right){ }$$

4 $${\text{P}} = \frac{E}{\Delta t} \times 3600 \left( {{\text{W}}/{\text{kg}}} \right),$$

where I is the discharge current, ∆t is discharge time, m is the mass of loaded MXene, and ∆V is sweep potential window. At 1 A/g current density, the C_g/cd_ for M1 and M4 without CVD-G were 32 and 228 F/g, whereas those with CVD-G are 52 and 254 F/g, and the corresponding specific energy densities were 4.44, 31.67 Wh/kg and 7.23, 35.28 Wh/kg, respectively. The maximum specific power densities for M1 MXene without and with CVD-G were 12.787 kW/kg and 16.268 kW/kg, whereas those for M4 were 14.252 kW/kg and 18.144 kW/kg, respectively. The charge–discharge curves of M2 and M3 MXene are shown in Figure S4 (e–h), and corresponding specific capacitances at 1A/g are in Table S4 (Supporting Information).

The resistance of the supercapacitors was estimated using EIS measurements, which are based on Nyquist impedance plots, performed at 10 mV sinusoidal signal in the frequency range of 100 kHz to 0.1 Hz (Fig. 4k). The Nyquist plots exhibited very low internal resistance, R_s_ (< 0.2 Ω), and almost vertical lines in the M4 MXene in both cases, which included with and without CVD-G, that indicated excellent capacitive character. However, it can be seen that the data without CVD-G show partial semicircles in the Nyquist plots for both the MXene at high frequencies, which imply some charge-transfer resistance (R_ct_) between the electrode and electrolyte indicating Faradaic reactions^47^. No semicircles are observed in the corresponding CVD-G based supercapacitors Nyquist plots, which indicates that the CVD-G mitigates the charge-transfer resistance. The very low resistance supports the charge/discharge curves, which show negligible voltage drop as previously discussed. The Nyquist plots of the M1 Ti_3_C_2_T_x_ MXene are not perfectly vertical, but they are slightly slanted, which may be ascribed to the diffusion impedance or the Warburg impedance (W), and it may possibly originate from the ions/mass diffusion at the electrode/electrolyte interface^48^. As observed from the EDX and the XPS studies, the M4 Ti_3_C_2_T_x_ MXene have more fluorides, which may have contributed towards the higher impedance. However, with the introduction of the CVD-G, the Nyquist plot of the M4 approaches a vertical line at lower frequencies, which indicates improved capacitive behavior^49^. Hence, it is clear that the CVD-G is not only improving the R_ct_ but also facilitating the mass transfer. A modified Randles equivalent circuit for these plots and the corresponding fitted plots obtained by the EC-Lab software and the equation are shown in Figure S5 (Supporting Information). The C_1_ and C_2_ are possibly the pseudo-capacitance by the faradaic process and the double-layer capacitance, respectively^17,47^. The fitted values of R_s_, R_ct_, C_1_, C_2_, and W for all supercapacitors are shown in Table S5 (Supporting Information). Based on the fitting results of the Nyquist plots, the limit capacitances of C_1_ are estimated as 106.4, 711.1, 127.2, and 800.0 F/g for the M1 and the M4 without and with CVD-G, respectively. It is confirmed from Table S5 that the parameters of the CVD-G based supercapacitors are superior without the CVD-G counterparts. The values of R_s_, R_ct_ and W are among the lowest reported values in the MXene based supercapacitors^17,19,21,50^. The low values of W in the order of magnitude of 0.1 Ω implies that the diffusion process of the ions is not significant. Judging from that the C_1_ is much greater than the C_2_ for all the devices, the pseudo-capacitive feature is dominant in our MXene based supercapacitors^42^. The specific capacitance of C_1_ for M4 with CVD-G is estimated as 800 F/g from this equivalent circuit fitting, which is a remarkably high value that is in agreement with the value roughly estimated from the CV. This difference in specific capacitance values obtained by CV plots and those obtained from equivalent circuit fitting is because CV curve based specific capacitance was calculated at 5 mV/s scan rate. This gap will be further minimized if the specific capacitance is measured at lower scan rate.

## Conclusions

In this study, two strategies were adopted to improve the supercapacitor properties using Ti_3_C_2_T_x_ MXene. Firstly, a superior current collector was obtained by passivating an Ni-foil with a CVD-G. Secondly, novel Ti_3_C_2_T_x_ MXene was extracted from the supernatant after etching Ti_3_AlC_2_ with LiF/HCl. These MXene have nano-layered structures. The synthesized MXene have some terminal groups, which were mostly -O and -F, as confirmed by using EDX and XPS analysis. The electrochemical properties of the supercapacitors that had different types of Ti_3_C_2_T_x_ MXene by repeated centrifuging were studied and compared. It was observed that the M4 MXene had almost 2–3 times the capacitance as compared to the M1 MXene in both cases with and without CVD-G. Also, the capacitance further improved to nearly double when the current collectors are passivated with graphene. A high specific capacitance, ~ 542 F/g has been achieved from CV measurement at 5 mV/s scan rate. The capacitance retention also improved significantly with the CVD-G passivation. The GCD curves of the supercapacitors are nonlinear, which indicates the pseudo-capacitive nature. The Nyquist plots of the M1 and M4 devices become close to vertical lines without semicircles by introducing CVD-G, which indicates that the CVD-G reduces the charge-transfer resistance from the electrode to electrolyte and improves the capacitive character. Hence, both strategies adopted in this study can open the myriads of opportunities in energy storage applications.

## Supplementary Information

Supplementary Information
