# Supplementary material for: Supercapacitors based on Ti3C2Tx MXene extracted from supernatant and current collectors passivated by CVD-graphene
Source: Sci Rep. 2021 Jan 12;11:649. doi: 10.1038/s41598-020-80799-9 (PMC7804397; doi:10.1038/s41598-020-80799-9)
Supplement: Supplementary file 1 — Supplementary Information [file 41598_2020_80799_MOESM1_ESM.docx]

**Supporting Information**

**Supercapacitors Based on Ti_3_C_2_T_x_ MXenes Extracted from Supernatant**

**and Current Collectors Passivated by CVD-Graphene**

Sunil Kumar^1,2^, Malik Abdul Rehman^1,2^ , Sungwon Lee^1^, Minwook Kim^1^, Hyeryeon Hong^1^,

Jun-young Park^1^, Yongho Seo^1,2*^

*^1^Department of Nanotechnology and Advanced Materials Engineering, Sejong University, Seoul, 05006, South Korea*

*^2^Graphene Research Institute and HMC, Sejong University, Seoul, 05006, South Korea*

*^*^Corresponding author: Email- yseo@sejong.ac.kr (Yongho Seo)*

- **FESEM and Raman spectra**


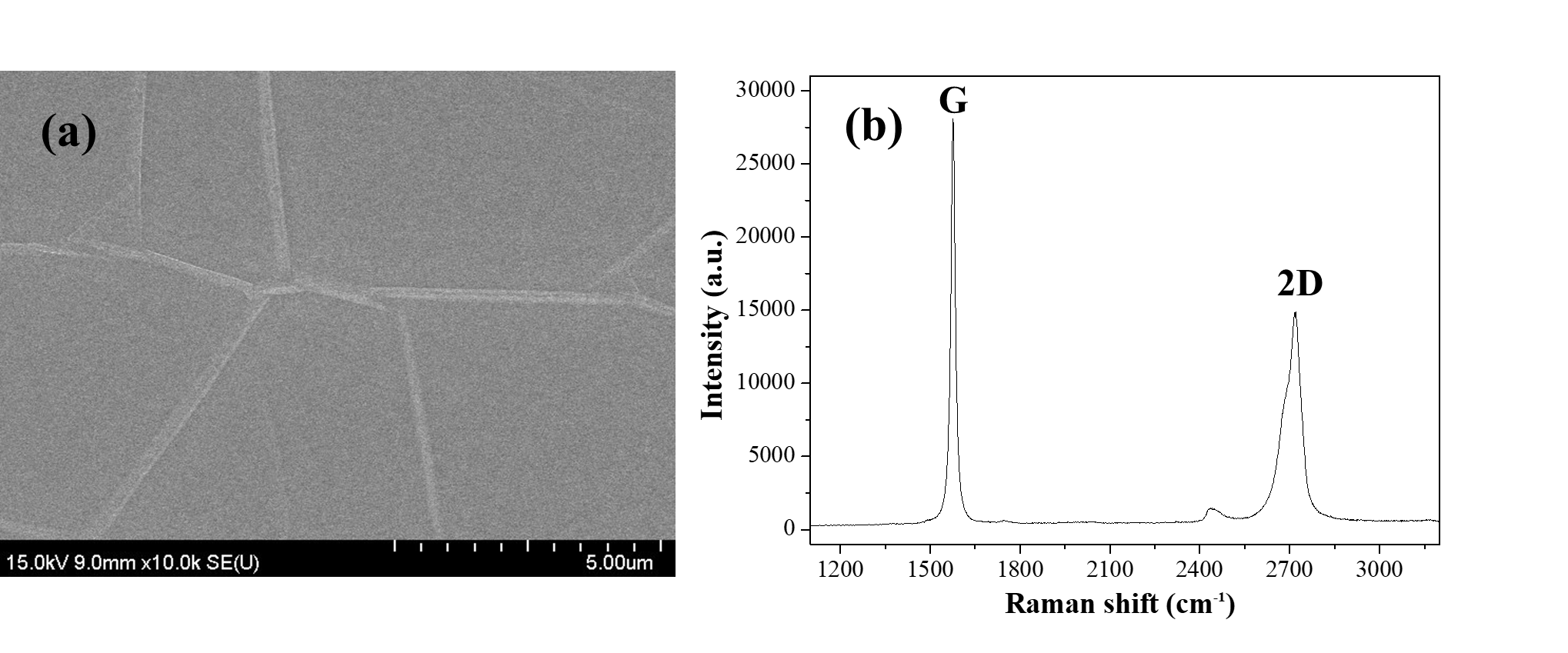


**Figure S1. (a) FESEM and (b) Raman spectra of CVD-graphene grown on Ni-foil.**

- **EDX study**

**Figure S2: (a) EDX spectrum of Ti_3_AlC_2_, (b-c) EDX spectra, (d-e) combined elemental maps of M1 and M4 MXene. Individual elemental maps of (f-k) M1 and (l-q) M4 MXene having Ti, C, O, F, Cl, and Al, elements, respectively.**

The EDX spectrum in Figure S2(a) shows the presence of Ti, Al, and C, corresponding to composition of Ti_3_AlC_2_ MAX phase. The EDX spectra in M1 and M4 MXenes indicate the presence of Ti, C, F, O, Cl, and Al elements. The individual element maps of Ti, C, F, O, Cl, and Al indicate the uniform distribution in M1 and M4 MXenes as shown in Figure S2(f-k) and S2(l-q), respectively.

**Table S1. EDX elements in Ti_3_AlC_2_**

| **Element** | **Wt.%** | **At. %** |
| --- | --- | --- |
| **C** | 32.59 | 33.24 |
| **Al** | 11.0 | 10.59 |
| **Ti** | 56.41 | 56.17 |
| **Total:** | 100.00 | 100.00 |

**Table S2. EDX elements in M1 MXene**

| **Element** | **Wt.%** | **At. %** |
| --- | --- | --- |
| **C** | 29.82 | 31.01 |
| **O** | 25.38 | 27.43 |
| **F** | 12.42 | 13.32 |
| **Al** | 0.89 | 0.59 |
| **Cl** | 1.95 | 1.48 |
| **Ti** | 29.54 | 26.17 |
| **Total:** | 100.00 | 100.00 |

**Table S3. EDX elements in M4 MXene**

| **Element** | **Wt.%** | **At. %** |
| --- | --- | --- |
| **C** | 25.48 | 32.30 |
| **O** | 22.76 | 26.19 |
| **F** | 19.70 | 19.96 |
| **Al** | 0.22 | 0.17 |
| **Cl** | 1.47 | 0.91 |
| **Ti** | 30.37 | 20.47 |
| **Total:** | 100.00 | 100.00 |

- **XPS analysis**

**Figure S3. (a-b) Complete XPS spectra of M1 and M4 MXenes are shown. (c-d) High-resolution XPS spectra of Cl and Al elements of M1 are shown. (e-f) High-resolution XPS spectra of Cl 2p and Al 2p peaks of M4 are shown.**

- **Cyclic voltammetry and charge discharge curves of M2 and M3 MXenes**


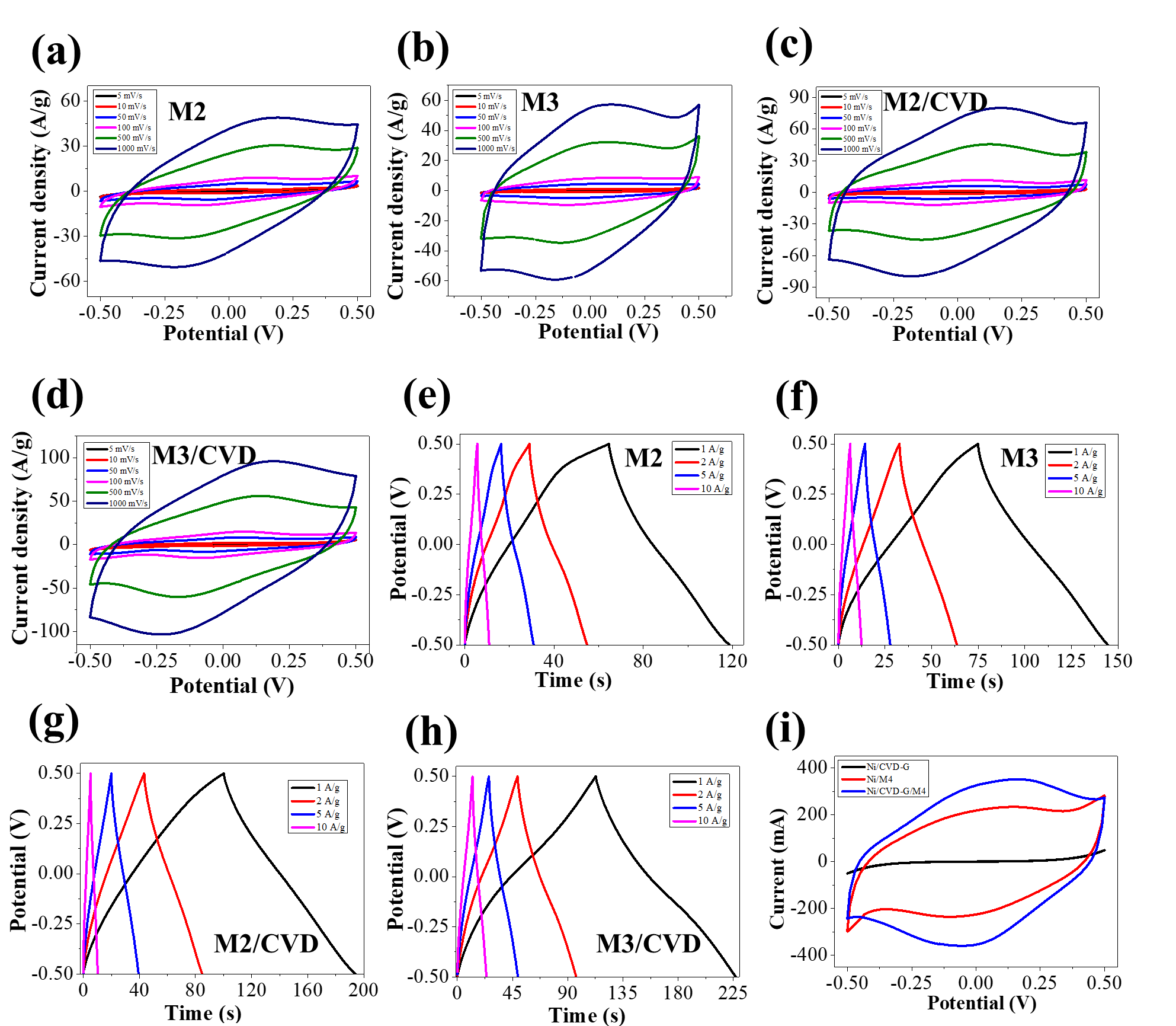


**Figure S4: (a-d) CV curves and (e-h) charge-discharge curves for M2 and M3 MXene, (i) CV curves of Ni/CVD-G, Ni/M4 and Ni/CVD-G/M4 at 1000 mV/s scan rate.**

**Table S4: The specific capacitance of all MXenes from CV and CD curves**

| **Electrode** | **C_g_ (F/g) @ 5 mV/s** | **C_g/cd_ (F/g) @ 1 A/g** |
| --- | --- | --- |
| **M1 MXene** | 116 | 32 |
| **M2 MXene** | 234 | 126 |
| **M3 MXene** | 281 | 138 |
| **M4 MXene** | 296 | 228 |
| **M1 MXene-CVD-G** | 156 | 52 |
| **M2 MXene-CVD-G** | 398 | 196 |
| **M3 MXene-CVD-G** | 459 | 224 |
| **M4 MXene-CVD-G** | 542 | 254 |

- **Nyquist plot fitting**

The Nyquist plots have been fitted using EC-Lab software. The closest equation fitted for the impedance (Z) corresponding to the Nyquist plots is:

$$Z= R_{s}+{i\omega C}_{1}+\frac{1}{\frac{1}{{i\omega C}_{2}}+ \frac{1}{R_{ct}+W}}$$

$= R_{s}+{i\omega C}_{1}+\frac{1}{\frac{1}{{i\omega C}_{2}}+\frac{1}{R_{ct}+\frac{A_{W}}{\sqrt{w}}+\frac{A_{W}}{i\sqrt{w}}}}$ ,

where R_s_ is series resistance, C_1_ is the double-layer capacitance, C_2_ is the faradaic capacitance, and W is Warburg impedance (W) = $\frac{A_{W}}{\sqrt{\omega}}+\frac{A_{W}}{i\sqrt{\omega}}$, where A_w_ is Warburg coefficient, $\omega$ is angular frequency.

**Figure S5. (a-d) Nyquist plots for M1 and M4 MXenes with and without CVD-G were fitted to (e) an equivalent circuit diagram using EC-Lab software (v11.33, BioLogic company,** [**https://www.biologic.net/ support-software/**](https://www.biologic.net/%20support-software/)**ec-lab-software/).**

| **Electrode** | **R_s_ (Ω)** | **R_ct_ (Ω)** | **C_1_ (F)** | **C_2_ (F)** | **W (Ω)** | **Mass (mg)** | **C_1_/g (F/g)** |
| --- | --- | --- | --- | --- | --- | --- | --- |
| **M1 MXenes** | **0.16** | **0.03** | **4.8** | **0.01** | **0.19** | **45.1** | **106.43** |
| **M4 MXenes** | **0.09** | **0.041** | **3.2** | **0.013** | **0.32** | **4.5** | **711.1** |
| **M1 MXenes-CVD-G** | **0.12** | **0.002** | **3.5** | **0.021** | **0.12** | **28.0** | **127.2** |
| **M4 MXenes-CVD-G** | **0.06** | **0.015** | **2.8** | **0.07** | **0.26** | **3.5** | **800.0** |

**Table S5. Fitting results for the equivalent circuit to Nyquist plots based EIS parameters**

**Figure S6.** **CV curves of (a) M1 MXene, (b) M4 MXene without CVD-G, (c) M1 Mxene with CVD-G, and (d) M4 Mxene with CVD-G, respectively, at 5 and 10 mV/s scan rates.**
